# Assessment of Patients' Future Outlook after Bowel Resection in Crohn's Disease

**DOI:** 10.1155/2019/7674946

**Published:** 2019-01-09

**Authors:** Shasha Tang, Xiaolong Ge, Weilin Qi, Wei Liu, Qian Cao, Wei Zhou

**Affiliations:** ^1^Department of General Surgery, Sir Run Run Shaw Hospital, School of Medicine, Zhejiang University, Hangzhou, China; ^2^Department of Gastroenterology, Sir Run Run Shaw Hospital, School of Medicine, Zhejiang University, Hangzhou, China; ^3^Inflammatory Bowel Disease Center, Sir Run Run Shaw Hospital, School of Medicine, Zhejiang University, Hangzhou, China

## Abstract

**Background:**

Many patients with Crohn's disease (CD) require surgery in their life. Their future outlook is crucial to psychological and mental health after surgery. This study is aimed at assessing CD patient's outlook after experiencing bowel resection and determining factors associated with patient's outlook.

**Methods:**

We used an ad hoc questionnaire (modified WHOQOL-BREF) to assess patients' outlook for the future after bowel resection in CD. All patients who experienced bowel resection for CD from 2015 to 2017 were included in this study. Patients were divided into two groups according to the questionnaire. Patients who had a positive outlook were compared with those who had a negative outlook. The patients' view on timing of surgery was also recorded.

**Results:**

Of 114 eligible patients surveyed, 103 (90.4%) responded. 65 (63.1%) reported that the timing of surgery was appropriate, and 26 patients felt it should have been performed earlier, while remaining 12 preferred a later surgery. 61 (59.2%) patients had a positive outlook of their future lives, while 42 patients had a negative outlook. Factors as the financial burden, employment status, patients' view on timing of surgery, and clinical recurrence were associated with patients' outlook. In the multivariate analysis, only clinical recurrence was an independent risk factor for patient's future outlook.

**Conclusion:**

From this survey, it is clear that most patients who underwent an elective bowel resection for CD are satisfied with their timing of surgery. Patients who have clinical recurrence carry a significant negative outlook for their future life. Postoperative management which focuses on preventing clinical recurrence may enhance patients' outlook for the future.

## 1. Introduction

Crohn's disease (CD) is an incurable inflammatory bowel disease characterized by a chronic, relapsing course [1]. It manifests a variety of symptoms, including abdominal pain, diarrhea, malnutrition, anal fistula, and systemic manifestations. CD may cause significant work disability and a large economic cost to patients and society [2]. Patients' self-perception of health and an increase of the burdens of daily life are also common effects. These factors impair patients' overall health-related quality of life (HRQoL). The use of specific instruments to measure HRQoL in patients with inflammatory bowel disease has confirmed that the impairment of HRQoL in CD is substantial [3].

Medical therapy may induce and maintain disease remission. Nonetheless, even in the era of biologics, patients with CD commonly require surgery owing to complications such as stenosis, fistula, or failure of the medical therapies [4, 5]. 40-50% of patients with CD will require bowel surgery at some time after diagnosis; up to 70% will require a second operation [6]. Unfortunately, Crohn's disease cannot be cured by surgery. The primary objective of surgery is to alleviate patients' symptoms and improve the HRQoL [7]. Previous studies have revealed that intestinal resection of all macroscopic Crohn's disease in patients treated with postoperative prophylactic drug therapy is associated with a significant and sustained improvement in HRQoL [8].

Psychological issues like patients' outlook are now recognized as having an impact on the course of inflammatory bowel disease (IBD), which also has a real negative impact on HRQoL [9]. Literature regarding CD patients' future outlook, especially those who experienced surgery, is lacking. A positive future outlook may serve as a resource to promote resiliency in contexts of adversity and to adapt with adverse life experiences and obtain positive outcomes [10]. Because CD is a life-long chronic, excruciating disease that can have devastating effect on patients' life, more attention should be paid to patients' future outlook. It is also important to investigate the related factors associated with the outcome of future outlook. Thus, the main objective of the present study was to evaluate the future outlook in a cohort of CD patients after surgery and to explore the demographic, personal, and social factors linked to future outlook. We also tried to provide some insight into patients' perspectives on timing of surgery in CD.

## 2. Materials and Methods

### 2.1. Study Population

Patients with CD who experienced an elective bowel resection from January 2015 to December 2017 were identified from a prospectively conducted IBD database. They were reviewed for eligibility for the study. In order to give participants time to fully recover after surgery, the study was limited to patients who had undergone surgery at least 3 months before the survey date. Patients with stoma, high-grade intraepithelial neoplasia, or CD-associated cancer were excluded. An online questionnaire regarding patients' outlook towards their future life was completed by patients. Informed consent was obtained from the patients, and the study was approved by the ethics committee of Sir Run Run Shaw Hospital.

### 2.2. Questionnaire and Assessments

A questionnaire designed for this survey was conducted online (WJX.cn). Participants were allowed to select one applicable option for most questions. We assessed future outlook using a specific validated WHOQOL-BREF questionnaire modified by us [11]. WHOQOL-BREF contains 26 items with five choices for each item. The five choices are incremented from 1 to 5, where 1 is total disagreement and 5 is complete agreement. To confirm the patients' choice clearly, at the end of the questionnaire, we added a question about attitude of his/her future outlook. Patients were also asked the question “Thinking about the future, how well do you agree with each of following? (I am sure I can handle work or school. I will have a happy life. I will have friends and people that care about me. My life will be interesting. My parents or children will be proud of me).” Responses were rated with a 2-point scale (1 = negative and 2 = positive). We also collected the patients' preference regarding the timing of the operation. Additionally, they were asked to provide sociological and economic details such as the education level, marital status, and financial burdens, along with demographics, clinical details, concurrent medications, sense of well-being, and so on. Medical data including disease location, disease phenotype, previous surgery, surgery procedure, indication of surgery, and postoperative complication and recurrence were obtained from our database. Preoperative disease phenotype and disease location were classified according to Montreal classification in 2005 [12]. Conventional therapy indicated mesalazine, glucocorticosteroids, thiopurines, or methotrexate. Endoscopic recurrence after surgery was scored using the Rutgeerts classification, of which i2, i3, or i4 disease was considered to have endoscopic evidence of recurrence [13]. Clinical recurrence was defined by a Harvey–Bradshaw index (HBI) score greater than 4 [14]. Survey data, including clinical and demographic data, were crosschecked with each patient's hospital records to ensure data accuracy.

### 2.3. Statistical Analysis

Results were expressed as mean ± SE or median (range) for continuous data, whereas categorical data were presented as number (%). For analysis, patients were categorized into the following 2 groups according to their outlook of the future life: group A, patients whose outlook of the future life was positive and group B, patients whose outlook was negative. Subsequently, differences between groups were analyzed regarding patient-related, disease-related, and surgery-related data by using the *χ*^2^ test, *t* test, and Mann-Whitney *U* test, as appropriate. Factors previously shown to influence the patients' outlook on univariate analysis were entered into a multivariate logistic regression analysis to compute odds ratios. Confidence intervals were set at 95%. A two-tailed *P* value of <0.05 was considered statistically significant. All statistical analysis was performed using SPSS software, version 18 (SPSS Inc., Chicago, IL).

## 3. Results

A total of 114 patients were eligible for the study, of which 103 (90.4%) responded. The average time from surgery to the survey was 22.8 (12.0-31.2) months. The characteristics of the study population are shown in Table 1. Of the 103 patients included in the analysis, 75 (72.8%) were male. 9 (8.7%) patients were less than 16 years old, 75 (72.8%) were between 16 and 40, and 19 (18.5%) were more than 40 years. 59 (57.3%) patients had a duration of disease of more than 5 years. 34 (33.0%) patients had previous intestinal resection. A total of 61 (59.2%) patients (group A) had positive future outlook for their future life, and 42 (40.8%) (group B) had negative future outlook in terms of patient choice on the modified WHOQOL-BREF questionnaire.

### 3.1. The Characteristics of Patients with CD

Comparison of baseline characteristics in patients who had positive outlook (group A) with those who had negative expectation (group B) showed no significant differences regarding gender, age at diagnosis, disease phenotype, disease duration, previous surgery, preoperative medication, smoking habit, place of living, and marital and educational statuses. However, the financial burden which was defined as being in debt or having no debt was significantly different between the two groups (Table 1). 64.3% of patients in group B were in debt, which was significantly more than that of patients in group A (44.3%, *P* = 0.046).

### 3.2. Perioperative Characteristics of Patients with CD

Concerning operative indications, no significant differences were observed between the two groups. Also, the two groups did not differ from each other regarding indications for surgery, surgical approach, postoperative complication, time since surgery and medication, and personal expense compared to the preoperative period (Table 2). In terms of medical expense, 46.6% of patients' expenses were decreased than before, while 31 (30.1%) stated higher than before and 24 (23.3%) remained similar. 90.2% of patients in group A returned to work postoperatively, while 69.0% of patients in group B did not, which was significantly different (*P* = 0.007). Furthermore, postoperative clinical recurrence was 33.3% in group B, while only 3.3% in group A, which was associated with patients' outlook (*P* < 0.001). Interestingly, 33 (32.0%) patients had endoscopic recurrence (Rutgeerts i2-i4) at survey time, which was not associated with a different future outlook compared with patients in endoscopic remission.

A total of 34 (33.0%) patients reported complications including wound infection, abdominal collection/abscess, anastomotic leak, and GI bleeding. Patients who experienced a postoperative complication did not demonstrate a significant influence on future outlook postoperatively. The future outlook outcome did not differ according to the surgical technique (open vs. laparoscopic).

### 3.3. Patients View on the Timing of Operation

65 (63.1%) patients reported that the timing of operation was appropriate, while 26 (25.2%) reported that the surgery should have been carried out earlier. The remaining 12 (11.7%) patients stated that they preferred a later operation or even no operation. All of these 12 patients had postoperative complications or clinical recurrence. Moreover, the patients' view on surgical timing was significantly different between group A and group B (*P* = 0.018), with more patients considering appropriate timing in group A (72.1%) than group B (50.0%).

### 3.4. Factors Influencing CD Patient's Expectation of the Future after Bowel Resection

In order to assess factors impacting the patients' outlook for the future life, multivariate analysis of relevant factors was performed as shown in Table 3. Only clinical recurrence (odds ratio = 0.081; 95% CI, 0.016-0.404; *P* = 0.002) was an independent risk factor for patients' outlook of future life. Other possible factors—financial burden, employment status, and patients' view on timing of surgery—were not significant factors for patient's outlook for the future life.

## 4. Discussion

In the present study, we analyzed the impact of clinical, sociodemographic, and economic variables on CD patients' future outlook after bowel surgery in a tertiary center. We found that clinical recurrence was a strong and independent risk factor for negative outlook in this CD cohort, and we demonstrate the importance of financial burden, patients' view on timing of surgery, and employment status for CD patients' future outlook after surgery.

Crohn's disease is a chronic, progressive, and destructive condition. It not only has a major impact on patients' HRQoL including relationships and employment but also has a huge impact on their expectation of future life, including a lack of confidence or hope [15]. Intestinal resection in patients treated with postoperative prophylactic drug therapy is associated with significant and sustained improvement in HRQoL [16]. Research has shown that ileocecal resection, both open and laparoscopic, is associated with significant improvement in HRQoL, at least in the short term [7, 17, 18]. Data investigating the outlook on future life for patients who have undergone bowel resection for CD is lacking despite evidence that a person's positive outlook of his or her future is important for health. The study sought to illuminate factors impacting patients' outlook for their future after bowel resection for CD. It is pleasing to know that more than half (59.2%) of patients who experienced bowel resection have positive outlook for their future.

In our survey, 33 (32.0%) patients had endoscopic recurrence, while 16 (15.5%) patients had clinical recurrence. It is surprising to learn that only clinical recurrence significantly decreased the outlook of patients' future, which is likely due to endoscopic recurrence usually having no symptoms for patients and the short follow-up time. Furthermore, the psychological impact is minimal [19]. These results are consistent with previous studies which have shown that postoperative clinical recurrence could reduce the HRQoL and confidence of patients, possibly leading to negative outlook [2].

In terms of pre- and postoperative economic expenses, most patients' annual expenses with CD were similar or decreased. However, there still were 54 (52.4%) patients having a financial burden (being in debt) after surgery, which was associated with patients' outlook. The financial burden may have resulted from surgery per se; as in the Markov's analysis, the estimated charges were higher for patients requiring surgery, but the duration of postsurgical remission was longer than that for medically treated patients [20]. Luo et al. [21] also have reported that costs of medical care strongly impair HRQoL in IBD. And the patient's economic burden caused by disease with CD may reduce patient's confidence and outlook to their future life. Therefore, socioeconomic support programs should be considered for integration into the management of IBD patients [21].

An intriguing finding is that although more than half of patients reported that timing of bowel resection was appropriate, a small proportion (25.2%) admitted that it should have been earlier. There is literature indicating that the timing of the initial consultation regarding the option of bowel resection in 87% of patients was appropriate [22]. However, there may be barriers to which delay surgery as treatment, including patients' fears or misconceptions about surgery [22]. CD patients may consider surgery as a failure or last resort. A recent multinational study has further demonstrated that surgery is one of the most feared concerns among patients from different countries [23]. Identifying and referring appropriate patients early for surgical consultation and providing the information better with regard to perioperative issues and potential complications may improve the patients' view on surgical timing and future outlook. Amazingly, 11.7% of patients felt that they have regretted performing operation. In these patients, 6 had postoperative complication and 6 had clinical recurrence. These may be the reasons for the negative appraisal of the surgical timing.

84 (81.6%) patients returned to work after bowel resection. Employment status was also related to patients' outlook for future life. It is well known that recovery working is possibly prompt to encourage CD patients who experienced bowel resection to facilitate psychological and mental health. Employment independently influences the overall disability in CD patients [24]. IBD patients without employment were with active disease, having lower HRQoL and higher anxiety and depression rates [25].

First limitation of the survey was that nonvalidated questionnaires were used to evaluate patients' outlook for future. We are aware that our ad hoc questionnaire did not address all the factors contributing to patients' outlook for future and its variability. But it was our intention to focus on how CD affects patients' outlook for the future postoperatively. Nevertheless, further work is required on the items and scales to fully complete the validation process, including confirmatory factor analysis utilizing a new sample. Secondly, the questionnaires were completed postoperatively which potentially introduced recall bias related to preoperative conditions. Thirdly, the small sample size of the study might have lacked the statistical power to detect differences between groups. Finally, this was a selected cohort of patients which was managed at a tertiary referral center; nevertheless, management options were discussed at multidisciplinary meetings. There was inherent referral and selection bias in terms of disease processes that led to the decision to operate. Further studies with a prospective design should be undertaken using validated questionnaires administered at different times after surgery. Studies looking at the effect of CD patients' outlook on the HRQoL are also needed.

In conclusion, most patients who underwent an elective bowel resection for IBD at our institution were satisfied with their timing of surgery. The financial burden, patients' view on timing of surgery, employment status, and clinical recurrence have effects on the patients' outlook; among which, clinical recurrence is a significant risk factor for patients' negative outlook after surgery. Postoperative management which focuses on preventing clinical recurrence may enhance patients' future outlook. The psychoeducational program is extraordinarily important in patients with negative outlook, for their ability to cope with the disease and as a help for their future life arrangement.

## Figures and Tables

**Table 1:** The characteristics of the study population.

Variable	All (*n* = 103)	Positive (*n* = 61)	Negative (*n* = 42)	*P* value
Gender, *n* (%)				0.244
Male	75 (72.8%)	47 (77.1%)	28 (66.7%)	
Female	28 (27.2%)	14 (22.9%)	14 (33.3%)	
Age at diagnosis (year)				0.467
A1 (≤16)	9 (8.7%)	5 (8.2%)	4 (9.5%)	—
A2 (17-40)	75 (72.9%)	47 (77.0%)	28 (66.7%)	—
A3 (>40)	19 (18.4%)	9 (14.8%)	10 (23.8%)	—
Location, *n* (%)				0.916
L1 (ileal)	35 (34.0%)	20 (32.8%)	15 (35.7%)	—
L2 (colonic)	7 (6.8%)	5 (8.2%)	2 (4.8%)	—
L3 (ileocolonic)	39 (37.9%)	23 (37.7%)	16 (38.1%)	—
L4 (upper gastrointestinal)	22 (21.3%)	13 (21.3%)	9 (21.4%)	—
Behavior, *n* (%)				0.920
B1 (nonstricturing/penetrating)	4 (3.9%)	2 (3.2%)	2 (4.8%)	—
B2 (stricturing)	70 (68.0%)	42 (68.9%)	28 (66.7%)	—
B3 (penetrating)	29 (28.1%)	17 (27.9%)	12 (28.5%)	—
Duration of disease (years)				0.233
<5	44 (42.7%)	29 (47.5%)	15 (35.7%)	—
≥5	59 (57.3%)	32 (52.5%)	27 (64.3%)	—
Smoker, *n* (%)	24 (23.3%)	18 (29.5%)	6 (14.3%)	0.073
Place of living, *n* (%)				0.971
Rural	37 (35.9%)	22 (36.1%)	15 (35.7%)	—
Urban	66 (64.1%)	39 (63.9%)	27 (64.3%)	
Marital status, *n* (%)				0.09
Single	30 (29.1%)	21 (34.4%)	9 (21.4%)	
Married	72 (69.9)	39 (63.9%)	33 (78.6%)	
Divorced	1 (1.0%)	1 (1.7%)	0 (0%)	
Education level, *n* (%)				0.151
Middle school or below	55 (53.4%)	29 (47.5%)	26 (61.9%)	—
College degree or above	48 (46.6%)	32 (52.5%)	16 (38.1%)	—
Financial burden, *n* (%)				0.046
Having no debt	49 (47.6%)	34 (55.7%)	15 (35.7%)	—
Being in debt	54 (52.4%)	27 (44.3%)	27 (64.3%)	—
Prior bowel resection, *n* (%)	34 (33.0%)	19 (31.1%)	15 (35.7%)	0.628
Medical therapy at time of surgery, *n* (%)				0.658
Conventional therapy	62 (60.2%)	36 (59.0%)	26 (61.9%)	—
Antitumor necrosis factor	24 (23.3%)	14 (23.0%)	10 (23.8%)	—
Other or no medicine	17 (16.5%)	11 (18.0%)	6 (14.3%)	—

**Table 2:** Perioperative characteristics of the study population.

Patient characteristics	All (*n* = 103)	Positive (*n* = 61)	Negative (*n* = 42)	*P* value
Indication for surgery, *n* (%)				0.216
Failure of medical treatment	6 (5.8%)	5 (8.2%)	1 (2.4%)	—
Bowel obstruction	66 (64.1%)	41 (67.2%)	25 (59.5%)	—
Fistula or abscess format	23 (22.3%)	11 (18.0%)	12 (28.6%)	—
Combination of these indications	8 (7.8%)	4 (6.6%)	4 (9.5%)	—
Laparoscopic surgery, *n* (%)	80 (77.7%)	50 (82.0%)	30 (71.4%)	0.207
Postoperative complication, *n* (%)	34 (33.0%)	20 (32.7%)	14 (33.3%)	0.954
Endoscopic recurrence, *n* (%)	33 (32.0%)	16 (26.2%)	17 (40.5%)	0.128
Time since surgery (months)	22.8 (12-31.2)	22.8 (12-30.2)	24 (13.3-30.6)	0.268
Current medical therapy, *n* (%)				0.336
Conventional therapy	52 (50.5%)	32 (52.5%)	20 (47.6%)	—
Antitumor necrosis factor	34 (33.0%)	18 (29.5%)	16 (38.1%)	—
Other or no medicine	17 (16.5%)	11 (18.0%)	6 (14.3%)	—
Employment status, *n* (%)				0.007
Employed	84 (81.6%)	55 (90.2%)	29 (69.0%)	—
Unemployed	19 (18.4%)	6 (9.8%)	13 (31.0%)	—
Annual personal expense, *n* (%)				0.488
Similar	24 (23.3%)	12 (19.7%)	12 (28.6%)	—
Higher than before	31 (30.1%)	18 (29.5%)	13 (31.0%)	—
Lower than before	48 (46.6%)	31 (50.8%)	17 (40.4%)	—
Clinical recurrence, *n* (%)/Harvey–Bradshaw				<0.001
Yes (>4)	16 (15.5%)	2 (3.3%)	14 (33.3%)	—
No (≤4)	87 (84.5%)	59 (96.7%)	28 (66.7%)	—
Patients' view on timing of surgery, *n* (%)				0.018
Earlier	26 (25.2%)	14 (23.0%)	12 (28.6%)	—
Later	12 (11.7%)	3 (4.9%)	9 (21.4%)	—
Appropriate	65 (63.1%)	44 (72.1%)	21 (50.0%)	—

**Table 3:** Factors influencing CD patients' outlook of the future after bowel resection.

Factors	OR	95% CI	*P* value
Financial burden	2.272	0.908-5.682	0.079
Employment status	0.318	0.095-1.061	0.062
Patients' view on timing of surgery	1.620	0.726-3.618	0.239
Clinical recurrence	0.081	0.016-0.404	0.002

## Data Availability

The data are included in our articles as tables and others which can be emailed by the first author (sst0903@163.com).

## Abbreviations

CD: Crohn's disease
HRQoL: Health-related quality of life
IBD: Inflammatory bowel disease
HBI: Harvey–Bradshaw index.
